# A Comparative Study of the Antibacterial, Antifungal and Antioxidant Activity and Total Content of Phenolic Compounds of Cell Cultures and Wild Plants of Three Endemic Species of *Ephedra*

**DOI:** 10.3390/molecules15031668

**Published:** 2010-03-11

**Authors:** Ali Parsaeimehr, Elmira Sargsyan, Katayoun Javidnia

**Affiliations:** 1 G.S.Davtyan Institute of Hydroponics Problems, National Academy of Sciences Republic of Armenia, Yerevan, 375082, Armenia; E-Mail: hydrop@netsys.am (E.S.); 2 Medicinal & Natural Products Chemistry Research Center, University of Medical Science Shiraz, Shiraz, 71345-3388, Iran; E-Mail: mncrc@sums.ac.ir (K.J.)

**Keywords:** *Ephedra*, callus culture, antibacterial, antifungal, antioxidant, phenol content

## Abstract

Investigations were carried out to determine antimicrobial and antioxidant properties and total phenol content of three wild species of *Ephedra* compared with their respective callus cultures. Callus induction was performed in a standard Murashige and Skoog (MS) medium with the following hormonal ranges (mg/L) for every species NAA:1.5, Kin:1 for *Ephedra strobiliacea*, NAA:2, Kin:1 for *Ephedra procera* and NAA:2, Kin:0.5 for *Ephedra pachyclada*. These ranges of PGPR (Plant Growth Promote Regulators) were chosen based on callus induction rates, RGR (Relative Growth Rate) and their fresh weights. An antimicrobial test against five Gram negative and two Gram positive bacteria and two fungi was performed using the disc diffusion method. All methanolic extracts showed antimicrobial activity, but the antimicrobial activity of the callus cultures was lower than those of the wild plants. *E. strobilacea* showed the highest antimicrobial activity, and all methanolic extracts of the wild plants and callus cultures unexpectedly showed the highest antimicrobial activity against *Pseudomonas aeruginosa*. A FRAP (Ferric Reducing Antioxidant Power) test was conducted to evaluate extracts for antioxidant activity. *E. strobilacea* with 1.61 ± 0.08 mmol eq quercetin/g extract and 0.278 ± 0.02 mmol eq quercetin/g extract for the wild plant and callus, respectively, showed the highest results. The total phenol content of extracts was measured by a Folin Ciocalteau test. All the chosen species displayed phenol contents but *E. strobilacea* had the highest amount (504.9 ± 41.51 μmol eq catechin/g extracts and 114.61 ± 15.13 μmol eq catechin/g extracts for the wild plants and callus, respectively).

## 1. Introduction

*Ephedra*, a medicinal plant belonging to the Ephedraceae Dum. Family, is a genus of non-floweringseed plants belonging to the Gnetales, the closest living relative of the Angiosperms [1,2]. *Ephedra* has been used for many years in Traditional Chinese Medicine to treat allergies, bronchial asthma, chills, colds, coughs, edema, fever, flu, headaches, and nasal congestion and has been a natural product source of alkaloids such as ephedrine, pseudoephedrine, norpseudoephedrine and other related compounds [3,4,5]. This prodigious plant also shows significant antimicrobial activity. There are reports concerning the biological activity of *Ephedra major* and its potential to inhibit growth of and AF (aflatoxin) production by *Aspergillus parasiticus* NRRL 2999 [6]. A decoction of *Ephedra* showed *in vitro* antibacterial activity against various bacterial species, including *Staphylococcus aureus, Bacillus anthracis, B. diphtheriae, B. dysenteriae, B. typhosus* and *Pseudomonas aeruginosa* and its volatile oil showed inhibitory activity against Asian influenza virus [7,8]. Kwon *et al.* [9] reported the antimicrobial activity of *Ephedra sinicia* extracts against bacteria, such as *Vibrio parahaemolyticus, Clostridium perfringens, Bacillus subtilis and Staphylococcus aureus*. In 2001 Caveney *et al.* [10], reported that *cis*-3,4-methanoproline, a cyclopropyl analogue of proline known to possess antimicrobial activity, was found in large amounts in the stems and seeds of many *Ephedra* species. An anti-yeast activity of different Iranian medicinal plants against three yeast species (*Saccharomyces cerevisiae*, *Candida albicans* and *C. utilis*) was reported and the antimicrobial effect of *E. intermedia* was confirmed [11]. The growth inhibition activity of materials derived from the stems of *E. pachyclada* against intestinal bacteria was examined, purification of the active constituent from *E. pachyclada* stems was performed and the active component was identified as quinaldic acid. The growth inhibiting activity varied according to the chemical compounds, dose, and bacterial strain. Quinaldic acid derived from *Ephedra* showed a strong inhibition against *Clostridium difficile* and *Clostridium perfringens* at 1.0 mg/disc, moderately inhibited the growth of *C. difficile* and *C. perfringens* at 0.5 and 0.1 mg/disc, but had no effect on the growth of *Bifidobacterium bifidum*, *Lactobacillus acidophilus*, and *Lactobacillus casei* [12].

Antioxidants are important in the prevention of human diseases. Antioxidant compounds may function as free radical scavengers, complexing agents for pro-oxidant metals, reducing agents and quenchers of singlet oxygen formation [13,14,15]. Antioxidants are often used in oils and fatty foods to retard their autoxidation; therefore, the importance of search for natural antioxidants has greatly increased in the recent years [16]. 

Plant-derived polyphenols are of great importance because of their potential antioxidant and antimicrobial proper ties. Phenolic compounds exhibit a considerable free-radical scavenging (antioxidant) activity, which is determined by their reactivity as hydrogen- or electron- donating agents, the stability of the resulting antioxidant-derived radicals, their reactivity with other antioxidants and finally their metal chelating properties [17,18].

According to the literature the callus has the potential to show secondary metabolite activity and can often be compared in this respect with the original plant. According to this aim the usage of cells could have industrial purposes by cells immobilization in a matrix for use in bioreactors. Besides the genetic potential of the donor plant for callus induction and the growth of callus in an *in vitro* culture, the choice of suitable PGPR and the medium is always considered to be as the most important step. MS medium is one of the most preferred media for callus induction and callus growth because of its sufficient nutrients [19]. There are several reports using MS medium for *Ephedra* callus induction. O'Dowd *et al.* [20] reported that in her experiments all nine chosen species of *Ephedra* producedcalluses on modified MS medium supplemented with 0.25 µM kinetin (Kin) and 5.0 µM 2,4-dichlorophenoxyacetic acid (2,4-D) or 1-naphthaleneacetic acid (NAA). 

Our experiments involved a comparative determination of three species of *Ephedra* wild plants and their callus cultures due to their potential to produce antibacterial, antifungal, antioxidant activity and their relation to total content of phenolic compounds.

## 2. Results and Discussion

### 2.1. Callus Induction

The following PGPRs (mg/L) were more effective for our chosen *Ephedra* species: NAA:1.5, Kin:1 for *Ephedra strobiliacea*, NAA:2, Kin:1 for *Ephedra procera* and NAA:2, Kin:0.5 for *Ephedra pachyclada* and they were therefore chosen for callus induction. Among all the species tested *E. procera* recorded significantly higher levels of 19.38 mg/explant of fresh weight and 237.7 ± 9.3 mg/mg/day of relative growth rate (Table 1). Depending on the chosen cytokines, Kin and BAP (6-benzylaminopurine), the responses to callus induction and fresh weight varied among treatments. Finally, we found that Kin was more effective in comparison with BAP and callus induction, and fresh weight and RGR were significantly increased when NAA was used as an auxin along with Kin as a cytokine in the three chosen *Ephedra* species.

Among the chosen species *Ephedra procera* subsequently recorded 6,650 ± 260 mg fresh weight (a 344% increase) and achieved the highest amount among other treatments after 112 days, this amount was 140.6 ± 21.6 mg fresh weight for *E. strobilacea* and 134.5 ± 12.4 mg fresh weight for *E. pachyclada*. O’Dowd *et al*. [20] reported that *E. major* Host. subsp. *Procera* achieved the highest fresh biomass compared with the other tested species. It is a known fact that callus growth and development are strongly related to genetic background and physiological status, the source of tissue, chemical compositions and physical state of the culture medium and culture conditions [21]. 

**Table 1:** Effect of chosen hormonal range for *Ephedra* species.

Plant	Induction rate (%)	Initial fresh weight (mg/ex)	Final fresh weight (mg)	RGR (mg/mg/day)
*E. procera*	68%	19.38 ± 0.82	6650 ± 260	237.7 ± 9.3
*E. strobilacea*	62%	17.36 ± 0.53	3920 ± 310	140.6 ± 21.6
*E. pachyclada*	52%	17.42 ± 0.39	3760 ± 270	134.5 ± 12.4

All the data are expressed as an average among 25 treatments in a triplicate factorial test.

### 2.2. Anti-Microorganism Activity

The comparative results of treatments for antimicrobial activity are presented in Table 2. According to our disc diffusion tests, the methanolic extracts of wild plants showed antimicrobial activity at all applied levels against the chosen microorganisms and their antimicrobial activity increased significantly at 4 mg/disc. Our results revealed that among three chosen *Ephedra* species, *E. strobilacea* showed the highest antimicrobial activity and achieved the highest activity index among treatments against most tested microorganisms and provided the most reliable antimicrobial activity of the three species of *Ephedra* against three microorganisms- *Pseudomonas aeruginosa* a Gram negative bacteria, *Staphylococcus aureus* a Gram positive bacteria and *Aspergillus nigra* a fungi. These results were also reported by the other experts [7,8,11].

Although the *E. strobilacea* showed the highest antimicrobial activity against most of chosen microorganisms regard to our test it seems that the plant methanolic extract of *E. pachyclada *significantly showed antimicrobial activity among the other chosen species against *Klebsiella pnemoniae* a Gram negative bacteria and *Bacillus subtilis* a Gram positive bacteria. Further more we realize the methanolic extract of *E. Procera* wild plant significantly showed highest antifungal activity against *Candida albicans* microorganism among the other treatments (Table 2).

Consider to our callus culture we found out that callus extraction displayed antimicrobial activity in lower ranges but by increasing the extracts mg/disc the inhibitions zone got significantly visible. We realize among three chosen *Ephedra* species, *E. strobilacea* callus extract showed the highest antimicrobial activity against most tested microorganisms (Table 2). Amazingly all methanolic extracts of *Ephedra* callus cultures showed antimicrobial activityagainst *Pseudomonas aeruginosa* (PTCC 1074) a Gram negative bacteria even in 0.5 mg/mL the lowest applied level. We found out *E. procera* methanolic extract expressed the lowest antimicrobial activities in callus culture among the other chosen species even against *Candida albicans* that its wild plant showed the highest antifungal activity. 

Literature reviews indicated that activation of phytoalexines produced from phenylproponanoieds metabolites were suggested for phenolic metabolites and could be expressed against pathogens which is strongly considered to limit the spread of the invading pathogens [15,17,22,25].

By determination the activity indices of extracts against chosen microorganisms were compared with their relative controls (Table 2), our data revealed that that the chosen species of *Ephedra* plants has antimicrobial activity against tested microorganisms in pharmaceutical usage. Although the activity index was significantly lower in callus extracts compared with extract obtained from plants, it also indicated that the callus had the potential to express the desired metabolites and this importance has been reported by other researchers too[23]. 

**Table 2:** Antimicrobial activity of wild plant and callus culture in three species of *Ephedra.*

Type of Microorganism		*E. procera*				*E.* *pachyclada*				*E. strobilacea*				Contol
**Gram Negative Bacteria**		**0.5**	**1**	**2**	**4**	**0.5**	**1**	**2**	**4**	**0.5**	**1**	**2**	**4**	
*Staphylococcus epidermidis* (PTCC 1114)	**EPIZ**	8.3 ± 1.5	9 ± 1.7	11.6 ± 0.5	13.3 ± 1.1	7.33 ± 1.5	10.6 ± 1.5	12 ± 1	14 ± 1	8.3 ± 0.5	9.3 ± 1.2	12.3 ± 0.5	15.3 ± 1.15	21 ± 3
	**EPAI**	0.41 ± 0.07	0.45 ± 0.08	0.58 ± 0.02	0.66 ± 0.5	0.38 ± 0.08	0.56 ± 0.08	0.63 ± 0.5	0.73 ± 0.05	0.39 ± 0.02	0.44 ± 0.05	0.58 ± 0.02	0.73 ± 0.05	
	**ECIZ**	-	-	8.6 ± 0.5	10 ± 1	-	-	8.6 ± 0.5	10 ± 0.5	-	-	8.6 ± 0.5	10 ± 1	24 ± 2
	**ECAI**	-	-	0.43 ± 0.02	0.45 ± 0.1	-	-	0.48 ± 0.03	0.55 ± 0.05	-	-	0.4 ± 0.04	0.58 ± 0.02	
*Escherichia coli* (PTCC 1338)	**EPIZ**	7.3 ± 1.5	9.6 ± 1.1	12 ± 1.7	14 ± 2	9.3 ± 1.1	10.3 ± 0.5	11.6 ± 0.5	13 ± 1	7.3 ± 1.5	8.6 ± 1.5	14 ± 1	15.6 ± 0.5	22 ± 3
	**EPAI**	0.32 ± 0.06	0.43 ± 0.05	0.54 ± 0.07	0.63 ± 0.09	0.46 ± 0.05	0.51 ± 0.02	0.58 ± 0.02	0.65 ± 0.05	0.33 ± 0.06	0.39 ± 0.02	0.63 ± 0.04	0.71 ± 0.02	
	**ECIZ**	-	-	-	-	-	-	9 ± 1.5	11.6 ± 1.2	-	-	9 ± 1	11.6 ± 0.5	22 ± 2
	**ECAI**	-	-	-	-	-	-	0.36 ± 0.07	0.53 ± 0.02	-	-	0.4 ± 0.02	0.52 ± 0.06	
*Salmonella typhi* (PTCC 1693)	**EPIZ**	6.6 ± 0.5	8 ± 1	11 ± 1	13.3 ± 1.1	9.3 ± 1.5	10.6 ± 1.15	11 ± 1	12.3 ± 1.5	7.6 ± 1.15	10 ± 1	13.6 ± 1.5	15.3 ± 1.15	22 ± 1
	**EPAI**	0.33 ± 0.02	0.4 ± 0.05	0.55 ± 0.05	0.66 ± 0.05	0.42 ± 0.06	0.48 ± 0.05	0.5 ± 0.04	0.56 ± 0.06	0.34 ± 0.05	0.45 ± 0.06	0.62 ± 0.06	0.69 ± 0.05	
	**ECIZ**	-	-	7.3 ± 0.5	8.6 ± 0.5	-	-	7.3 ± 0.5	8.6 ± 1.2	-	-	7.3 ± 0.5	8.3 ± 0.5	21 ± 3
	**ECAI**	-	-	0.46 ± 0.07	0.56 ± 0.07	-	-	0.34 ± 0.02	0.37 ± 0.02	-	-	0.32 ± 0.02	0.38 ± 0.04	
*Pseudomonas aeruginosa* (PTCC 1074)	**EPIZ**	10 ± 1	12.6 ± 1.1	15.3 ± 0.5	17.3 ± 1.1	12.6 ± 1.1	14.3 ± 1.15	15.6 ± 1.5	17.3 ± 1.15	10.3 ± 1.52	11.3 ± 1.15	15.3 ± 0.5	18 ± 1.15	24 ± 2
	**EPAI**	0.41 ± 0.04	0.52 ± 0.04	0.63 ± 0.02	0.72 ± 0.04	0.6 ± 0.05	0.68 ± 0.05	0.72 ± 0.02	0.74 ± 0.07	0.43 ± 0.06	0.47 ± 0.02	0.63 ± 0.02	0.82 ± 0.05	
	**ECIZ**	9 ± 1.5	10.6 ± 1.5	12.3 ± 0.5	13.3 ± 1.52	7.6 ± 0.5	10.6 ± 1.1	12.3 ± 0.5	13.6 ± 1.5	9 ± 1	10.61 ± 0.5	12.3 ± 0.5	13.6 ± 1.5	22 ± 4
	**ECAI**	0.38 ± 0.06	0.44 ± 0.06	0.52 ± 0.04	0.59 ± 0.02	0.36 ± 0.02	0.53 ± 0.07	0.56 ± 0.06	0.64 ± 0.07	0.35 ± 0.02	0.41 ± 0.03	0.51 ± 0.02	0.59 ± 0.02	
*Klebsiella pnemoniae* (PTCC 1031)	**EPIZ**	7.6 ± 1.1	9 ± 1	11 ± 2	13.3 ± 1.1	6.3 ± 0.5	9.3 ± 0.5	10.3 ± 1.1	14 ± 0.5	7.3 ± 1.5	8 ± 1.2	10.3 ± 1.1	12.3 ± 1.5	23 ± 2
	**EPAI**	0.31 ± 0.04	0.37 ± 0.04	0.45 ± 0.08	0.55 ± 0.04	0.35 ± 0.03	0.51 ± 0.03	0.57 ± 0.06	0.77 ± 0.03	0.3 ± 0.02	0.33 ± 0.02	0.43 ± 0.04	0.51 ± 0.06	
	**ECIZ**	-	-	-	-	-	8.3 ± 1.5	9 ± 1	12.3 ± 0.5	-	-	8.3 ± 1	11 ± 1	24 ± 2
	**ECAI**	-	-	-	-	-	0.36 ± 0.02	0.44 ± 0.03	0.55 ± 0.05	-	-	0.34 ± 0.02	0.45 ± 0.04	
**Gram Positive Bacteria**														
*Bacillus subtilis* (PTCC 1023)	**EPIZ**	-	8.3 ± 2.5	10 ± 1.7	12 ± 2	8.6 ± 1.1	10 ± 1	13 ± 1	16 ± 2	8.6 ± 2	9.6 ± 1	12 ± 0.5	12.3 ± 0.5	24 ± 1
	**EPAI**	-	0.34 ± 0.1	0.41 ± 0.07	0.5 ± 0.08	0.37 ± 0.05	0.43 ± 0.04	0.56 ± 0.04	0.69 ± 0.61	0.37 ± 0.09	0.42 ± 0.06	0.52 ± 0.5	0.53 ± 0.06	
	**ECIZ**	-	-	-	10.6 ± 1.52	-	-	8 ± 2.6	10.6 ± 1	-	-	10.3 ± 0.5	10.6 ± 1.5	23 ± 2
	**ECAI**	-	-	-	0.38 ± 0.06	-	-	0.41 ± 0.02	0.43 ± 0.04	-	-	0.32 ± 0.04	0.46 ± 0.02	
*Staphylococcus aureus* (PTCC 1112)	**EPIZ**	-	11.3 ± 1.1	14.6 ± 1.1	16.3 ± 1.5	8.6 ± 2	12.6 ± 1.5	14.6 ± 1.1	16 ± 2	9.6 ± 1	10.3 ± 0.57	14.3 ± 0.5	17.3 ± 1.1	24 ± 1
	**EPAI**	-	0.45 ± 0.04	0.58 ± 0.06	0.65 ± 0.06	0.36 ± 0.08	0.52 ± 0.06	0.61 ± 0.04	0.66 ± 0.08	0.38 ± 0.04	0.41 ± 0.03	0.57 ± 0.02	0.69 ± 0.04	
	**ECIZ**	-	8.6 ± 1	10.3 ± 0.5	11.6 ± 0.5	-	8.6 ± 1.5	10.3 ± 0.5	11.6 ± 1	-	8.6 ± 0.5	10.3 ± 0.5	11.6 ± 0.5	22 ± 2
	**ECAI**	-	0.36 ± 0.04	0.38 ± 0.02	0.45 ± 0.02	-	0.38 ± 0.02	0.44 ± 0.05	0.46 ± 0.02	-	0.37 ± 0.04	0.41 ± 0.02	0.46 ± 0.06	
**Fungi**														
*Aspergillus nigra* (PTCC 5010)	**EPIZ**	-	8.3 ± 2.5	12.6 ± 1.1	14.6 ± 1.1	9.6 ± 0.5	11.6 ± 1.5	12.3 ± 1.5	16 ± 2	-	9 ± 1	14.3 ± 0.5	16.3 ± 1.52	25 ± 2
	**EPAI**	-	0.33 ± 0.1	0.5 ± 0.04	0.58 ± 0.04	0.37 ± 0.02	0.44 ± 0.05	0.47 ± 0.05	0.61 ± 0.07	-	0.37 ± 0.04	0.59 ± 0.02	0.68 ± 0.06	
	**ECIZ**	-	9 ± 1	10.3 ± 0.5	11.6 ± 0.5	-	9.3 ± 1.1	11 ± 0.05	11.3 ± 0.5	-	9 ± 1	10.6 ± 0.5	11.3 ± 1.5	25 ± 1
	**ECAI**	-	0.36 ± 0.04	0.41 ± 0.02	0.46 ± 0.02	-	0.35 ± 0.04	0.42 ± 0.04	0.43 ± 0.04	-	0.37 ± 0.04	0.43 ± 0.02	0.46 ± 0.02	
*Candida albicans* (PTCC 5027)	**EPIZ**	7.6 ± 1.5	9 ± 2	14.6 ± 1.1	16.6 ± 1.1	10.3 ± 1.5	12 ± 2	13.3 ± 1.15	14.6 ± 1.1	-	8.3 ± 0.5	11.6 ± 0.5	12.6 ± 1.15	23 ± 2
	**EPAI**	0.41 ± 0.06	0.48 ± 0.08	0.53 ± 0.04	0.58 ± 0.04	0.4 ± 0.06	0.36 ± 0.04	0.58 ± 0.4	0.66 ± 0.04	-	0.33 ± 0.06	0.46 ± 0.02	0.5 ± 0.04	
	**ECIZ**	-	-	-	-	-	-	8 ± 1	9.6 ± 1.1	-	8.3 ± 1.1	10.3 ± 0.5	12.3 ± 0.5	25 ± 1
	**ECAI**	-	-	-	-	-	-	0.41 ± 0.02	0.49 ± 0.02	-	0.32 ± 0.04	0.44 ± 0.02	0.49 ± 0.04	

All data were given as the average between three separated tests. Mean value n = 3 ± standard error.EPIZ: *Ephedra*'s plant extract inhibition zone (mm) including the diameter of disc (6 mm) ; EPAI: *Ephedras* plant extract activity index = Inhibition area of test sample/inhibition area of the standard ; ECIZ: *Ephedras* callus extract inhibition zone(in mm) including the diameter of disc (6 mm) ; ECAI: *Ephedras* callus extract activity index=Inhibition area of test sample/inhibition area of the standard ; - : Not detected ; Control : the standard 10 µg/ disc that Gentamicin was used for Gram negative bacteria, Ampicillin was used for Gram positive bacteria and Ketoconnazole was used for fungi.

### 2.3. Antioxidant Activity Ferric Reducing Antioxidant Power (FRAP) Assay

The antioxidant tests based on a FRAP assay are presented in Table 3. These results revealed that the wild plants and callus of all three chosen *Ephedra* species had antioxidant activity, but the callus showed lower activity than wild species. It also indicated that among three species of *Ephedra* the mehanolic extract of *E. strobilacea* showed the highest activity compared to the other species in three separate factorial tests with a good coefficient of variation (cv 10.32) with an amount of 1.61 ± 0.08 mmol eq quercetin/g extract for wild plant and 0.278 ± 0.02 mmol eq quercetin/g extract for callus in three replicated tests (cv:13.44). 

**Table 3:** Wild plant and callus culture antioxidant comparing efficiency of three species of *Ephedra* based on FRAP assay.

Varients	*E. procera*	*E.* *pachyclada*	*E.strobilacea*
Wild Plant	1.6 ± 0.09*/ 6.90 ± 0.71**	1.56 ± 0.05* /6.96 ± 0.71**	1.61 ± 0.08*/6.96 ± 0.63**
Callus culture	0.248 ± 0.02*/1.19 ± 0.15**	0.275 ± 0. 01*/1.18 ± 0. 87**	0.278±0.02* /1.19 ± 0.11**

All the data are expressed as *mmol eq quercetin/g extracts, **mmol eq FeSO_4_/g extracts, [mean value ± standard error (n = 3)].

In our second test using FeSO_4_ as a control (Table 3) the methanolic extract of *E. strobilacea* showed the highest antioxidant activity with 6.96 ± 0.63 mmol eq FeSO_4_/g extract for wild plant and 1.19 ± 0.11 mmol eq FeSO_4_/g extract for callus. The results obtained with FeSO_4_ as a control can be another confirmation of the antioxidant activity of the extracts. Antioxidants are broadly defined as molecules that when present at low concentrations, compared to those of oxidizable substrates, significantly delay or prevent oxidation of that substrate. The antioxidant potential of *Ephedra* extract seems to be due to its strong hydrogen-donating and metal chelating ability, as well as to its effectiveness as a scavenger of hydrogen peroxide and free radicals. Many studies have demonstrated the radical scavenging properties of plant phenolic compounds and confirm the relationship between phenolic compounds and antioxidant activity [24,25,26].

### 2.4. Phenol Content

The results of the Folin Ciocalteau test for determining the phenolic content are presented in Table 4. According to this test both wild plants and callus contained phenols and among them *E. strobilacea* wild plant showed the highest amount with 504.9 ± 41.51 μmol equivalent catechin/g extract (cv: 13), and its callus achieved the highest result of 114.61 ± 15.13 μmol equivalent catechin/g extract (cv: 13.2) too. We found out there was a strong relationship among total phenol content and antioxidant and antimicrobial activity, as phenols are very important plant constituents because of their scavenging ability on free radicals due to their hydroxyl groups. Therefore, the phenolic content of plants may be contributed directly to their antioxidant action, phenylpropanoid and flavonoid biosynthesis [17,27,28].

**Table 4:** Comparison of total phenol content in wild plant and callus culture of three*Ephedra* speciesbased on the Folin Ciocalteau method.

Varients	*E. procera*	*E.* *pachyclada*	*E.strobilacea*
Wild Plant	436.44 ± 59.14	454.5 ± 62.24	504.9 ± 41.51
Callus culture	105.56 ± 17.65	108.77 ± 17.15	114.61 ± 15.13

All the data are expressed as (μmol eq catechin/g extracts) [mean value ± standard error (n = 3)].

## 3. Experimental

### 3.1. Preparation of Plants Samples

Three species of *Ephedra*, *E. procera*, *E. strobilacea***,** *E. pachyclada* were collected from N29º23' E53º10' and identified by experts of the Medicinal & Natural Products Chemistry Research Center, University of Medical Science Shiraz, Iran for our aims, the callus induction and study the potential of *Ephedra* callus for using against microbial infections, antioxidant activity and as a source of phenolic compounds in pharmaceutical applications. 

### 3.2. Preparation of the Extracts

The aerial parts of the three species of *Ephedra *were dried in room temperature (26 ± 3 ºC) in the dark, and powdered. The methanolic extracts were obtained by maceration of the crude plant powder with methanol/water 90/10 for 2 days in a chamber temperature (26 ± 3 ºC) in the dark. The extracts then were filtered using a sterile cloth sheet and dried under reduced pressure at temperature below 45 ºC. Three species of *E. procera*, *E. *s*trobilacea*, *E. pachyclada* callus (obtained from various ranges of PGPRs), were freeze dried and used for antimicrobial activity after callus stabilization in the medium (6 months). The callus was extracted with methanol for 48 hours and methanol extracts were grouped from each callus culture medium and the solvent evaporated under vacuum on a rotary evaporator (below 40 ºC). Then the extracts were diluted in methanol to the appropriate range and analyzed for their inhibition zone (loaded µg/disc), antioxidant (mmol eq quercetin/g extract; mmol eq FeSO_4_/g extract) and total phenol (μmol eq catechin/g extracts) content.

### 3.3. Callus Induction

Stems were surface sterilized in sodium hypochlorite (0.5%) containing a few drops of Tween 20 for 7 min and rinsed five times with distilled water. Subsequently 0.8 ± 0.2 cm internodes were separated from the donor plant under a laminar flow hood and were again surface sterilized in sodium hypochlorite (0.25%) containing Tween 20 for 1 min and rinsed three times with distilled water. The wounded parts exposed to sterilization agent were trimmed and the healthful shoot tips were used as explants in the experiment. Finally 25 explants per treatment were used and monitored during days. 

A standard MS medium (Murashinge and Skoog, 30 mL in every flask) with various plant growth promote regulators such as 1-naphthaleneacetic acid (**NAA**) as an auxin and 6-benzylaminopurine (**BAP**) and kinetin (**Kin**) as cytokines with the following ranges: 0.5; 1; 1.5; 2 mg/L, respectively, were used for callus induction with 30 gr/L sucrose and 7 gr/L agar. The pH of the medium was adjusted to 5.8 using 1 N NaOH and 1 N HCL before autoclaving. Subsequently the explants which showed callus induction were kept under light (2,500–3,000 lux) at 24 ºC ± 2 for tests. Subcultures were conducted every four weeks and continued until callus stability. One hormonal range was chosen for every three species of *Ephedra* after the 4^th^ subculture based on the callus observation, fresh and dry weight, phenotype, callus stability in the medium and their RGR-s. The other samples were deleted subsequently after 4th subculture and just the samples with following ranges (mg/l) *E. procera* NAA:2 Kin: 1; *E. *s*trobilacea*; NAA:1 Kin:1; *E. pachyclada,* NAA: 1.5 Kin: 0.5 were monitored, reserved, subcultured and prepared for tests. Relative Growth Rate (RGR) was measured for the species using following formula:

RGR = 3(Wf⅓ - Wi⅓) / tf-ti

where Wi: callus initial mass (at ti), Wf: final callus mass (at tf), t = time, tf-ti = 28 days of subculture period.

### 3.4. Disk Diffusion Method

In each test, microorganisms were cultured at 37 ºC for 16–24 h and prepared to turbidity equivalent to McFarland standard No. 0.5. Then the suspensions were spread on the test plates (nutrient agar). Sterile discs were impregnated with 0.5, 1, 2 and 4 mg of the wild plants or callus extract, and placed on surface of test plate. Positive control discs with gentamicin, ampicillin and ketoconazole (10 µg/disc) for Gram negative bacteria, Gram positive bacteria and fungi, respectively, were run and compared afterwards with both calluses and plant extracts. Each extract and control was tested in triplicate and the experiments were repeated four times. The plant extracts were monitored against seven bacterial strains and two fungi strains at four concentrations of 0.5, 1, 2, 4 mg/L. The tested organisms were: *Escherichia coli* (PTCC 1338), *Bacillus subtilis* (PTCC 1023), *Staphylococcous aureus *(PTCC1112), *Staphylococcus epidermis* (PTCC 1114), *Pseudomonas aeruginosa *(PTCC 1074), *Aspergillus nigra* (PTCC 5010), *Candida albicans *(PTCC 5027), *Klebsiella pnemoniae *(PTCC 1031), *Salmonella typhi* (PTCC 1693) which were obtained from the Persian Type Culture Collection (PTCC), Medicinal & Natural Products Chemistry Research Center, University of Medical Science, Shiraz, Iran. 

### 3.5. Antioxidant Activity

The FRAP assay was carried out as a method described by Benzie and Strain (1996) [29] with slight modifications. The FRAP reagent was prepared by mixing anhydrous sodium acetate (38 mM) in distilled water (pH 3.8), FeCl_3_·6H_2_O (20 mM) in distilled water and 2,4,6-tri-(2-pyridyl)-*S*-triazine (TPTZ, 10 mM) in HCL (40 mM) in a proportion of 10:1 to each sample; appropriately diluted sample extract (100 µL) and FRAP reagent (900 µL) were added and the mixture incubated at room temperature for 30 min under a sodium lamp. In the case of the blank, methanol (100 µL) was added to FRAP reagent (900 µL). The absorbance of the resulting solution was measured at 593 nm by spectrophotometer [29]. The quercetin and FeSO_4_ obtained from Merck and prepared at a concentration of 0.1 mM were used as reference antioxidant standards. FRAP values were expressed as mmol eq quercetin/g extract and mmol eq FeSO_4_/g extract of the samples.

### 3.6. Determination of Total Phenolic Content

A Folin Ciocalteau test in triplicate was conducted for determination of total phenolic compounds and test was repeated three times. A protocol described by Singleton and Rossi (1965) [30] was used for our purpose. Plant extract (200 µL) was dissolved in methanol (mg/mL) and was placed into glass tubes in triplicate, then catechin (200 µL) provided by Sigma was dissolved in methanol (5 mg/20 mL) and used as a standard and Folin Ciocalteau (diluted 10 times, 2,500 µL) was added to the extracts and the mixture incubated at room temperature for 5 min and vortexed at least two times. Finally Na_2_CO_3_ (7.5% dissolved in water, 2,000 µL) was added and the tubes were closed with Parafilm, covered with aluminum foil and put on a shaker at 60 rpm for 90 min and the absorbance of samples measured at 760 nm [30]. The phenol content of the extracts was expressed as μmol eq catechin/g extract. Total phenol concentration was determined by using the formula:

(As/Ac) × (Cc/Sc) × 1000 × R

Where: As: sample absorbance; Ac: catechin absorbance; Cc catechin initial concentration; SC: Sample initial concentration; R: yield of callus or plant extracts.

### 3.7. Data Analysis

The obtained data were expressed as the average between the replicates [(mean value ± standard error (n = 3)] and the coefficient of variation (C.V) among the replication were analyzed using SAS software for accuracy and precision.

## 4. Conclusions

This paper attempts to present a comparative study of the antibacterial, antifungal, antioxidant activities and the total phenol contents of *Ephedra* wild plantsand their respective calluses. Our results clearly showed that the callus of three chosen species of *Ephedra* had the potential to produce the desired antibacterial, antifungal, antioxidant and phenolic metabolites but it seemed that the undifferentiation of cells in *in vitro* culture could be a reason for expressing these desired secondary metabolites in lower ranges compared with extracts which were provided from organs. Furthermore, besides wild plants genotype, they are exposed to a wide range of environmental stresses and these stresses most of the time indicated the increase of secondary metabolites. Nevertheless our results indicated the ability to utilize plant biotechnology techniques towards development of desired bioactive metabolites in *in vitro* culture instead of using wild plants in pharmaceutical purposes. 
